# High acceleration quiescent-interval single shot magnetic resonance angiography at 3T in patients with peripheral artery disease

**DOI:** 10.1186/1532-429X-15-S1-O55

**Published:** 2013-01-30

**Authors:** Parag Amin, Maria Carr, Marie Wasielewski, Jeremy Collins, Robert R Edelman, James Carr

**Affiliations:** 1 Radiology, Northwestern Memorial Hospital and Northwestern University Feinberg School of Medicine, Chicago, IL, USA; 2 Radiology, NorthShore University HealthSystem, Evanston, IL, USA

## Background

Quiescent-Interval Single Shot (QISS) non-contrast MR angiography (QISS-MRA) is an emerging technique with growing evidence for its use in the evaluation of peripheral artery disease (PAD) at 1.5T [1,2]. The use of 3 Tesla MRI scans is becoming more common in clinical practice and offers the main advantage of roughly double the signal-to-noise ratio (SNR) than that of 1.5 Tesla MRI. Improvements in B1 field inhomogeneity along with fast (parallel) imaging make QISS-MRA a viable technique on state-of-the-art 3T scanners. A prior study from this group investigated the potential to accelerate imaging at 3T using higher parallel imaging acceleration (PAT) factors on healthy volunteers [3]. With this technique optimized, we report preliminary results in clinical patients. The objective of this study is to evaluate the diagnostic quality of QISS at 3T in patients with PAD using contrast-enhanced MR angiography (CE-MRA) as the reference standard.

## Methods

With IRB approval, eight patients (5 males, age 58-78 yrs; 3 females age 37-82) with symptoms of chronic lower extremity PAD were recruited prospectively and underwent imaging on a 3T scanner (MAGNETOM Skyra; Siemens Healthcare, Germany) using an ECG-gated QISS-MRA sequence (PAT factor of 3); CE-MRA was acquired at 3T for each patient at the time of visit. The degree of stenosis was evaluated using a 5-point scale for 31 predefined arterial segments (from infra-renal aorta through to bilateral run-off vessels) on both QISS- and CE-MRA. The sensitivity and specificity of QISS-MRA at 3T for the determination of clinically significant (≥50%) versus non-significant (<50%) stenosis were calculated with CE-MRA as the reference standard; time-resolved acquisitions were included in the analysis.

## Results

QISS-MRA acquisitions were diagnostic for all patients, across all arterial segments. Vascular segment-based analysis demonstrated a sensitivity of 95.7% (44 of 46 segments) and a specificity of 98% (195 of 199 segments) for QISS-MRA using CE-MRA as the reference standard. Venous signal did not limit segment-based analysis on any of the QISS-MRA scans but was a confounding factor in the calf regions of 2 CE-MRA scans.

## Conclusions

Preliminary results from QISS-MRA at 3T demonstrate excellent performance with near complete agreement with CE-MRA in a small cohort of patients with chronic lower limb ischemia. Work is ongoing to validate these findings in a larger prospective cohort.

## Funding

Dr. Edelman has active funding from an R01 Grant from the NIH.

Dr. Collins has funding from the SIR Foundation and RSNA Research & Education Foundation.

**Figure 1:**
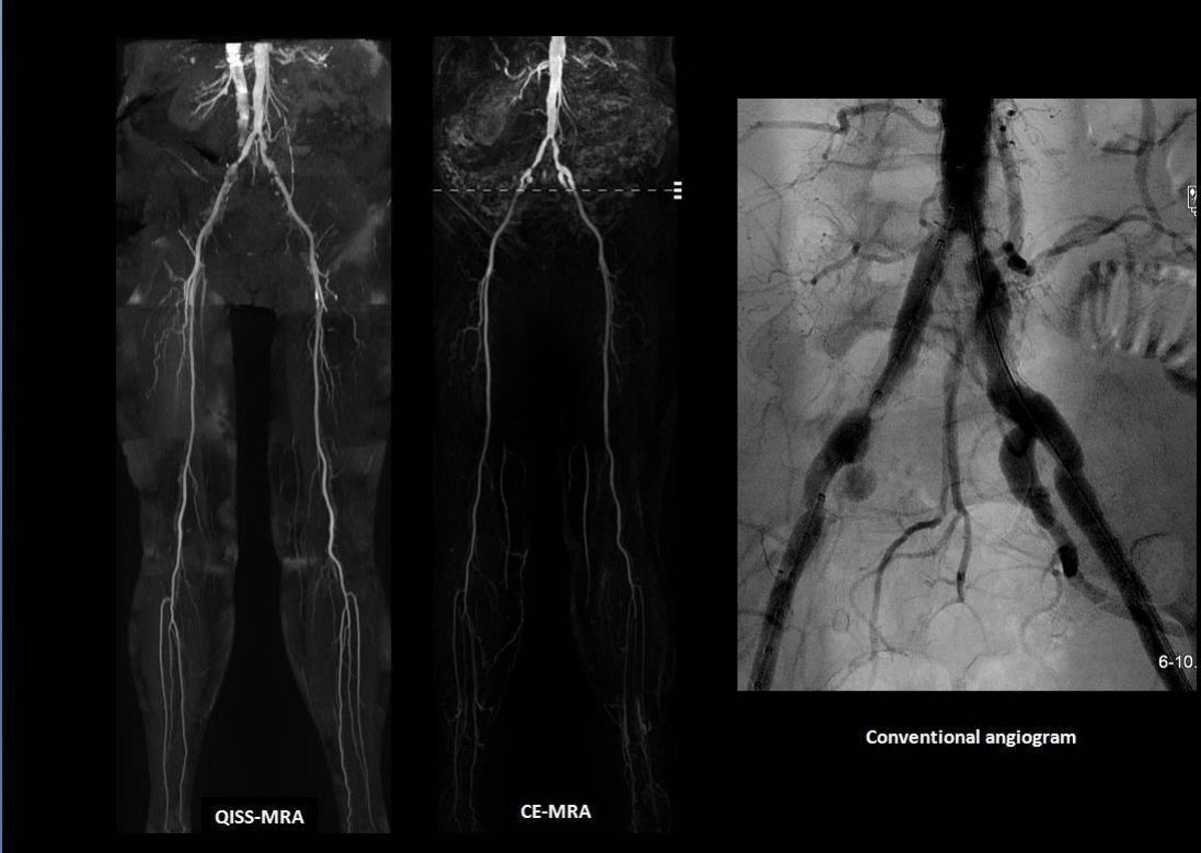
Representative patient case. 80-year old female with bilateral claudication with imaging findings of inflow disease in the iliac arteries. Note the dilution effect peripherally on CE-MRA vs consistent signal on QISS.
